# Adjustments of Speed and Path when Avoiding Collisions with Another Pedestrian

**DOI:** 10.1371/journal.pone.0089589

**Published:** 2014-02-26

**Authors:** Markus Huber, Yi-Huang Su, Melanie Krüger, Katrin Faschian, Stefan Glasauer, Joachim Hermsdörfer

**Affiliations:** 1 Center for Sensorimotor Research, Institute for Clinical Neuroscience, Ludwig-Maximilians-University, Munich, Germany; 2 Institute of Movement Science, Technical University of Munich, Munich, Germany; 3 Munich Center for Neurosciences – Brain and Mind, Ludwig-Maximilians-University, Munich, Germany; 4 German Center for Vertigo and Balance Disorders, Ludwig-Maximilians-University, Munich, Germany; 5 Bernstein Center for Computational Neuroscience Munich, Munich, Germany; University of Muenster, Germany

## Abstract

When walking in open space, collision avoidance with other pedestrians is a process that successfully takes place many times. To pass another pedestrian (an interferer) walking direction, walking speed or both can be adjusted. Currently, the literature is not yet conclusive of how humans adjust these two parameters in the presence of an interferer. This impedes the development of models predicting general obstacle avoidance strategies in humans’ walking behavior. The aim of this study was to investigate the adjustments of path and speed when a pedestrian is crossing a non-reactive human interferer at different angles and speeds, and to compare the results to general model predictions. To do so, we designed an experiment where a pedestrian walked a 12 m distance to reach a goal position. The task was designed in such a way that collision with an interferer would always occur if the pedestrian would not apply a correction of movement path or speed. Results revealed a strong dependence of path and speed adjustments on crossing angle and walking speed, suggesting local planning of the collision avoidance strategy. Crossing at acute angles (i.e. 45° and 90°) seems to require more complex collision avoidance strategies involving both path and speed adjustments than crossing at obtuse angles, where only path adjustments were observed. Overall, the results were incompatible with predictions from existing models of locomotor collision avoidance. The observed initiations of both adjustments suggest a collision avoidance strategy that is temporally controlled. The present study provides a comprehensive picture of human collision avoidance strategies in walking, which can be used to evaluate and adjust existing pedestrian dynamics models, or serve as an empirical basis to develop new models.

## Introduction

When walking in a shopping center or in a train station, pedestrians usually cross their paths with dozens of other people without colliding into them. To avoid collisions, two principal movement parameters have to be coordinated: the walking path and the speed. Here, walking *path* refers to the spatial parameter (i.e., changes in position) of a pedestrian’s trajectory, regardless of the temporal evolution. Walking *speed* refers to tangential velocity along the planned path, that is, independent of the movement direction. Although the combination of these two parameters allows for infinite possibilities to avoid collisions with obstacles, the observed movements appear to exhibit stereotypical trajectories within and across people [1] [2]. This indicates that pedestrians use specific strategies to avoid obstacles while moving towards their intended locations. In principal, while the adjustment of path can always lead to successful obstacle avoidance, the adjustment of speed alone may not be sufficient. Imagine a person standing in your way or approaching you in a head-on encounter: collision avoidance in this case cannot be achieved without changing the path.

The question of whether pedestrians adjust movement path or speed to avoid collision with another person has been of recent scientific interest, but results thus far have been inconsistent. In the presence of static objects, obstacle avoidance is mainly explained by path adjustment. This adjustment seems to be governed by the information about the person’s own movement in relation to the objects in the scene, namely, the distance to the goal, the distance to the static obstacle, as well as the obstacle’s angle with respect to the heading direction, as proposed by Fajen and Warren [3]. Recent work by Fajen and Warren [4] extents this model to moving obstacles. Further empirical evidence comes from Moussaid et al. [5], who reported that when passing a static human, people simply change their movement direction to avoid collisions when passing a non-moving human. On the other hand, adjusting the speed has the advantage of keeping the intended path so that a spatial re-planning of the movement trajectory is not necessary. Thus, braking seems to be favored when the field of view is restricted [6], in small areas, or crowded places [7], and when the environment or the obstacle’s behavior is uncertain [1]. Furthermore, Cinelli and Patla [8] report that braking is the only option in the presence of spatiotemporal restrictions, such as when passing through an oscillating door.

Besides these findings favoring either path or speed adjustment as a collision avoidance strategy, a number of studies also found adjustments in both parameters. Cinelli and Patla [9], for example, showed that when a human doll directly approached a walking person, the person changed its movement path prior to its walking speed. By contrast, Olivier et al. [10] reported a collision avoidance behavior for a moving obstacle crossing at an angle of 90°, starting with an adjustment of speed followed by an additional path adjustment. As the two studies provide inconsistent results regarding the order of initiation of path and speed adjustments, it seems that the opted obstacle avoidance strategy is highly dependent on the environmental constraints and the dynamics of the obstacle.

**Figure 1:**
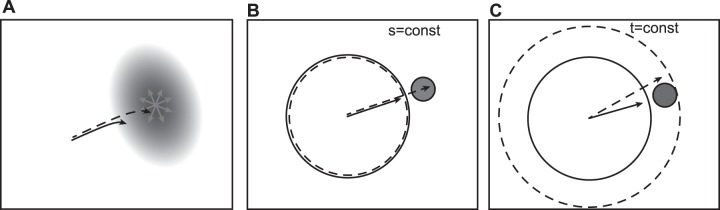
Illustration of model predictions about the influence of the obstacle distance on the avoidance strategy. The dashed arrows symbolize a fast walking speed of the pedestrian, the solid arrow a slower speed. **A:** The obstacle is represented as a repulsive potential. The fast speed allows the pedestrian to climb up the potential higher before the repulsive force leads to a significant change of the path as compared to a slow walking speed. **B:** A fixed spatial horizon (dashed and solid circle) specifies when an obstacle (grey circle) is taken into account. The horizon is not dependent on the speed (dashed and solid arrows) of the pedestrian [3] [7]. **C:** The horizon is speed dependent. Therefore, the obstacle is taken into account at a greater distance for a higher walking speed. This is comparable to a time-to collision mechanism [15].

Inconsistency of human collision avoidance strategies exists not only in the empirical data, but also in the models that attempt to describe this behavior. These models differ not only in their basic assumptions but also in their predictions about the deployment of path or speed adjustments in collision avoidance. To exemplify this, let us assume a scenario without considerable spatial constraints, and with only one moving obstacle (another person) walking at different speeds. Furthermore, the crossing person (i.e. the obstacle) does not react to the pedestrian by any means. A navigation model based on the heuristics [7] proposes that, as a first rule, a pedestrian chooses a walking direction that allows for the most direct path towards the goal, taking into account the obstacles in between. This model [7] uses a default maximum “horizon distance” of a pedestrian, for which obstacles are taken into account. Critically, this default “horizon distance” leads to adjustments of the path at a fixed distance to the obstacle, independently of the pedestrian’s walking speed (see Fig. 1B). A second rule of this model describes that the pedestrian tries to keep a minimal safety distance to the obstacles, which becomes relevant only within small distances to the obstacle or in the presence of spatial constraints. Given enough space to navigate, together the two rules converge to an adjustment of the path rather than of the speed. Other models inspired by Newton’s laws of motion (e.g., the “social force model” [11], or a modification of it [12]) describe the pedestrian’s motion as a combination of a driving force pointing towards the goal position, and repulsive forces originating from the obstacles. Due to the repulsive and driving forces, adjustments of the walking path are expected rather than adjustments of the walking speed. These models predict that path and speed adjustments are initiated closer to the interfering person when the pedestrian moves at a higher speed (see Fig. 1A). A third approach, based on the theories of optimal control, suggests that the smoothness of the movement is optimized [13]. In an improved version of this model, a penalty for speed changes was introduced [14], favoring path adjustments rather than speed adjustments in collision avoidance. As another approach, Fajen and Warren [3] modeled an obstacle avoidance behavior, in which the angular acceleration was described as a function of the goal, the obstacle angle and distance, taking into account only path adjustments, and ignoring speed changes, thereby predicting path adjustments at the same distance to an obstacle (see Fig. 1B). A last possible collision avoidance strategy, known as the time-to-collision strategy [15], proposes that the initiation of path and speed adjustments starts at larger distance to the obstacle with higher walking speed of the pedestrian, while the time point for the adjustment remains the same (see Fig. 1C).

In addition, different models predict different positions where static or moving obstacles become relevant. While models dealing with static obstacles mainly derive the collision avoidance behavior from the position of the obstacle [3], it is not yet clear which position of a moving obstacle is used as a cue. The “social force model” assumes a potential around the current position of the moving obstacle, which is shifted to the moving direction of the pedestrian, as the “pedestrian requires space for the next step” [11]. Other models pose that the pedestrians estimate possible collision points in the future and correct their trajectories according to their predictions [7] [12]. As previous empirical studies varied in the experimental conditions and environmental constraints, it is difficult to compare the existing data with different model predictions.

The goal of this study is to investigate obstacle avoidance strategies in several different pedestrian-obstacle constellations, and to compare the results with principle model predictions. Specifically, we investigated whether and how pedestrians, walking at different speeds, adjusted their movement path and speed in the presence of a human obstacle who crossed at different angles without reacting to the pedestrian’s behavior. Note that the behavioral variables, which guide these adjustments were not within the scope of this study, but rather how the pedestrians actually perform the adjustments of walking path and velocity. Different crossing angles were included, as we intended to examine its influence on the applied collision avoidance strategy. Furthermore, we aimed to reveal whether adjustments in both path and walking speed are jointly or independently controlled in space. Lastly, by analyzing different model predictions we intended to establish which position of the moving obstacle is taken into account to avoid a collision.

## Methods

### 1 Ethics Statement

The experiment was approved by the local ethics committee of the Ludwig-Maximilians-University Munich in accordance with the Declaration of Helsinki. Written informed consent was obtained from all participants prior to the experiment.

### 2 Participants

Ten healthy participants (6 female, mean age ± SD: 22.5±2.5 years) took part in this study and were paid for their participation. All had normal or corrected-to-normal vision, with no history of motor disease or impairment. A confederate experimenter (male, age 25) served as a human obstacle (*interferer*, see 3, below).

### 3 Experimental Setup and Procedure

Using an optical motion tracking system (Vicon Motion Systems, UK) the shoulder and clavicle positions (left and right acromion, and midpoint between the two clavicles, respectively) of the participants and interferer were tracked at 250 Hz using infrared-reflective markers. The tracked area was a 6×6 meter square in the middle of a room with approximately 70 m^2^. All experiments were done with eyes open and under natural lighting condition.

**Figure 2:**
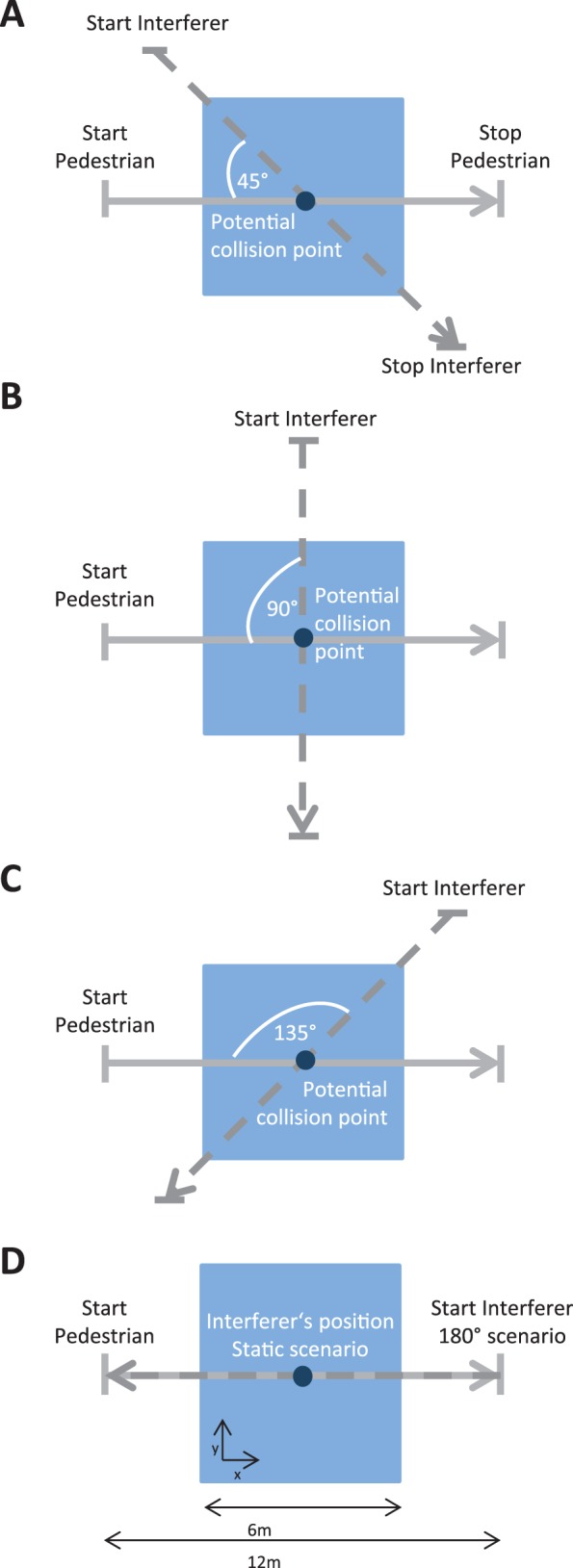
Experimental set-up. Each subfigure illustrates one of the different crossing scenarios. The blue square represents the tracked area. Further, the start and end positions of both participant and interferer, as well as the potential collision points are depicted. The walking direction of the participant is marked as grey arrow; the path of the interferer is marked as dashed arrow. **A:** 45° scenario. **B:** 90° scenario. **C:** 135° scenario. **D:** Static and 180° scenario.

Before the start of the experiment, the participants were instructed about the experimental task. Participants had to walk a total distance of 12 meters, of which the middle six meters were within the tracked area and which was predefined by a start and a stop position along the X-axis (see Fig. 2). The start and stop positions were chosen such that the participants entered and left the tracked area at a constant speed. They were informed that during their walk another pedestrian (the interferer), who would not react to them, might cross their path. Participants were told not to communicate verbally with the interferer. In total, six conditions (hereinafter called *scenarios*, see Fig. 2) were tested, which varied in the interferer’s behavior. The scenarios were presented in a blocked manner. In each block, the interferer could be absent or present depending on the scenario:

- Scenario (1) – No-interferer (baseline condition) – The participants walked the given distance in the absence of the interferer.
- Scenario (2) – Static-interferer – The participants walked the given distance while the interferer remained static in the middle of the straight path between start and stop position, facing the start position of the participant.
- Scenario (3) - 45°-interferer – The participants walked the given distance, while the interferer walked from the left of the participant in a straight path that crossed the middle of the participant’s original path at 45°;
- Scenario (4) - 90°-interferer – similar to (3), with the interferer crossing at an angle of 90°;
- Scenario (5) - 135°-interferer – similar to (3), with the interferer crossing at an angle of 135°;
- Scenario (6) - 180°-interferer – The interferer walked a straight path towards the starting position of the participant.

In addition, three *speed conditions* were included, in which the participants were instructed to walk: (1) in their natural speed, (2) in a speed that was faster than natural, and (3) in a speed that was slower than natural. An analysis of the mean speeds confirmed that the participants walked according to these instructions (see Results).

The interferer was trained to walk with a constant speed that matched the individual walking speed of the participant when entering the tracked area. In this way, he would intersect the participant in the middle of the tracking area if there would be no collision avoidance by the participant. The interferer estimated the intersection based on the walking speed of the participant during the initial 3 meters before entering the tracked area and walked accordingly in this speed throughout the trial. This was the case even if the participant did initiate any path and speed changes, which would of course render the initially estimated intersection invalid. Furthermore, he was instructed not to initiate any collision avoidance himself, even if a participant would not adjust his walking path or speed. Hence, he was told to be “non-reactive” to the participant’s behavior.

The experiment followed a 6 (scenario)×3 (speed) within-subject design, with the order of conditions being randomized across participants. Each scenario×speed combination was repeated five times, leading to 90 trials in total per participant.

### 4 Data Processing and Analysis

#### 4.1 Data processing

For each participant, the recorded trajectories of the three markers were first preprocessed offline in the Vicon Nexus 1.7.1 software (Vicon Motion Systems, UK). As soon as a marker was registered by at least two cameras, the position of the marker was recorded in the data. Labels for the markers were reattached if they were temporarily missing in the recorded trajectory. The preprocessed data were subsequently exported as planar XY coordinates without gap filling and were further analyzed using customized Matlab scripts (Mathworks, Natick, USA).

#### 4.2 Filter and interpolation

The position data was filtered using a Gaussian low pass filter (cutoff frequency 0.5 Hz) and a 5^th^ order media filter, as commonly used in other studies [9] [3] [16] [10]. These filter parameters were chosen to conserve the walking path as accurately as possible and simultaneously filtering the trunk oscillations caused by the steps [17]. Gaps in the recordings up to a duration of 0.2 sec were interpolated using the customized Matlab function ‘interp1’ with the shape-preserving piecewise cubic interpolation (‘pchip’) method.

**Figure 3:**
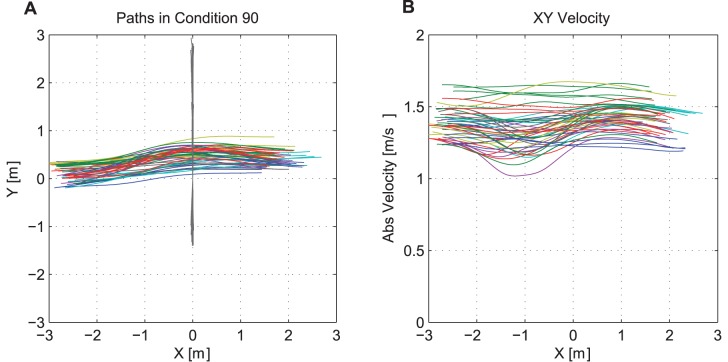
Example of the trajectories and speed profiles after applying the filter and coordinate system transformation. **A:** The plot shows an example of the recorded trajectories in the 90° scenario with normal walking speed. The participants are color-coded, the interferer’s trajectories is grey. **B:** Plot of the absolute speed along the movement direction.

To obtain the trajectories of the trunk, we calculated the geometrical average position using the two shoulder marker. This position dataset was compared to the dataset from the clavicle marker. The dataset with the most valid position entries was chosen as trunk position dataset. Figure 3 displays an example of the trajectories and velocities of the participants in a 90° scenario after applying the filter.

#### 4.3 Coordinate system transformation

We applied a coordinate system transformation to minimize the spatial variability of the interferer across trials and participants. To do so, a least square fit of the interferer’s trajectory was calculated. The difference of this angle and the predefined scenario angle was used to rotate the dataset. Please note that the rotation was applied to both, the interferer and the respective participant, thus preserving the spatial relation between them.

Additionally, for each trial we calculated a *point of minimal distance* (PoMD), which we defined as position on the participant’s trajectory where the participant reached the minimum distance to the interferer. Following that, we transformed the dataset so that the PoMD became the new point of origin of the coordinate system. This allowed us to characterize subsequent spatial parameters of the trajectories with regard to each individual’s point of minimal distance.

#### 4.4 Data analysis

**Figure 4:**
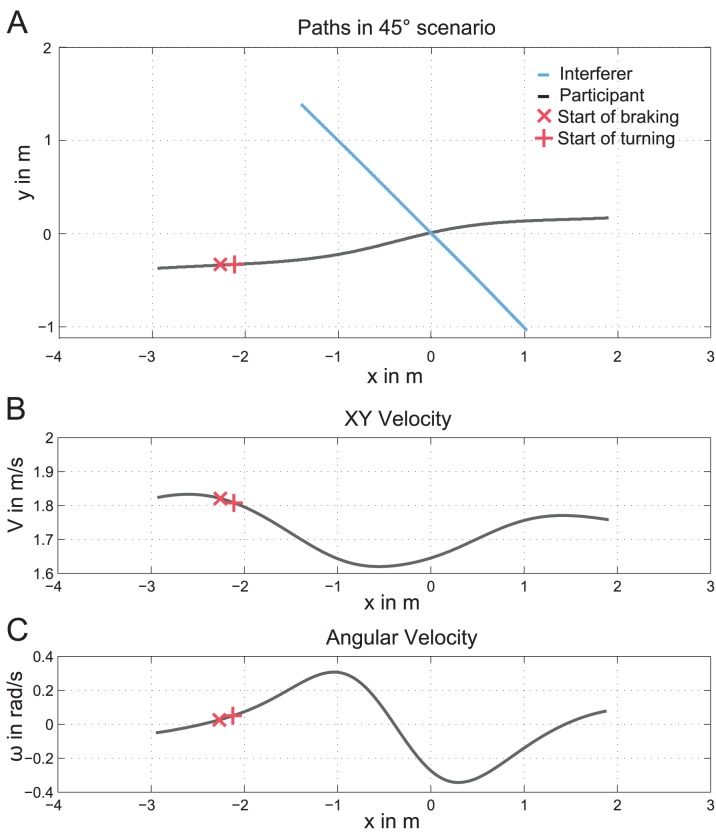
Illustration of the parameter of interest. The position of the start of turning is printed as +, the start of braking is shown as ×. **A:** Plot of the trajectory of a participant in the 45° scenario. **B:** Plot of the respective speed profile with respect to the point of minimal distance (PoMD). **C:** Plot of the angular speed as the distance to PoMD. The positions of the parameters are marked.

To be able to investigate path and speed adjustments, we defined a number of parameters, which will be explained in detail in the following. As a first general parameter, the walking direction was defined as the straight line from the start position to the stop position, which matched the X-axis of the coordinate system. Further, the current movement direction α_i_ was defined as angle of the tangent of the trajectory at position *i*:
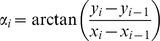



In addition, the angular speed ω_i_ was defined as the rate of change of the movement direction:
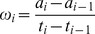



Please note that all path parameters refer to the distance to PoMD. All speed parameters refer to the absolute speed, which is the Euclidean norm of the speeds in x and y direction. Fig. 4 displays an example of a trajectory, speed and angular speed for a participant in the 45° scenario.

#### Path parameters

**Figure 5:**
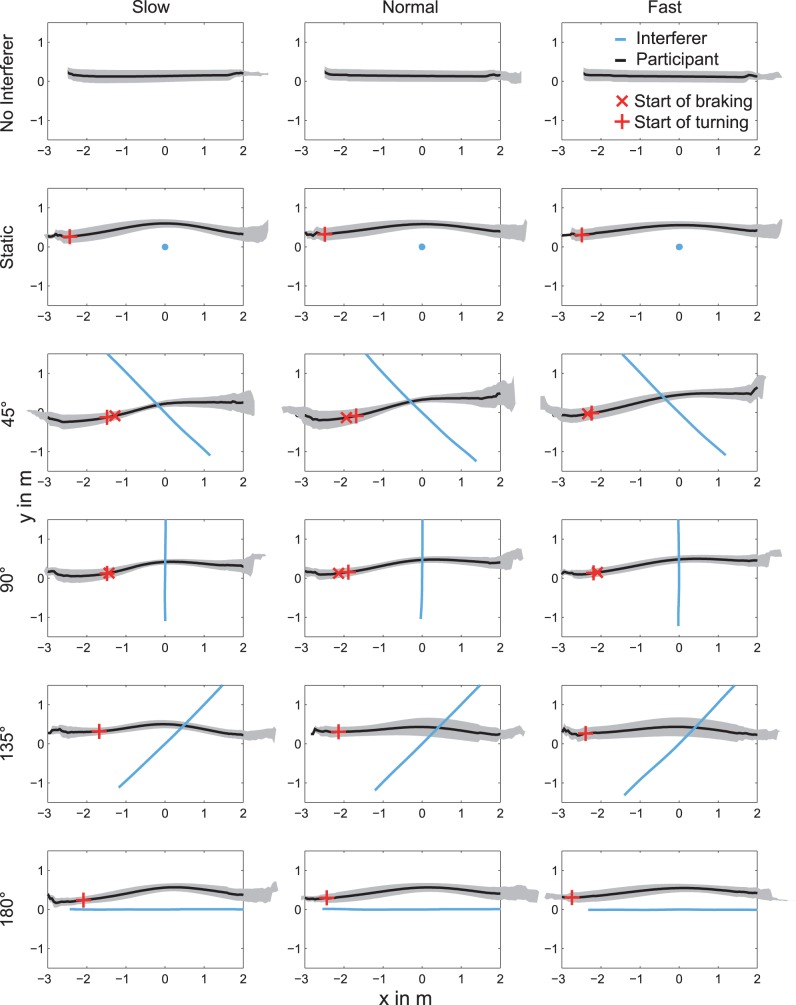
Plot of the mean trajectory across all participants. The standard deviation is displayed as grey area. The columns represent the speed conditions, the rows the scenarios. The interferer is represented as gray line (45°–180° scenarios) or dot (static scenario). The start of the speed adjustment (if present) is shown as ×, the start of the path adjustment is printed as +.

First, the *mean trajectory* along the walking direction was calculated. All trajectories were interpolated to 400 equally spaced points along the X-axis of the transformed coordinate system in the range from −3 m to 3 m with respect to the PoMD. With this procedure we were able to calculate an overall mean trajectory across participants for each scenario (see Fig. 5). Trials where the participant passed in front of the interferer (45°, 90° & 135° scenarios, 3 out of 900 trials in total), or where the participant passed towards the negative Y-direction (i.e. the interferers left side; static & 180° scenario) were not considered in this analysis.

Secondly, in order to measure the degree of spatial circumvention before participants passed the interferer, we calculated the *maximal lateral deviation* from the straight path, separately for each trajectory. The magnitude of displacement indicated how much the participants deviated from the straight path in order to avoid colliding with the interferer.

Thirdly, to determine the start position of the path adjustment (hereafter called the *start of turning*) we calculated the spatial distance between the point where the participant started to turn and the PoMD. To do so, we first calculated a baseline turning magnitude, separately for each participant and speed condition. The baseline turning magnitude represents the mean maximal angular speed in the No-interferer scenario across all trials. Subsequently, we calculated the start of turning for the remaining scenarios, which was defined as the first point where the magnitude of angular speed started to exceed the baseline turning magnitude. Since negative values were obtained, corresponding to points on the X-axis prior to PoMD, the values will be reported as absolute spatial distance to PoMD. An example of the start of turning is depicted as+in Fig. 4.

#### Spatial parameters

The *mean absolute speed* was calculated similar to the calculation of the mean trajectory. We first calculated the absolute speed at each point of the trajectory as the absolute value of the first derivate of the trajectory with respect to time. The absolute speed was then interpolated to 400 equally spaced points along the walking direction in the range from −3 m to 3 m with respect to PoMD. Subsequently, we performed the averaging along the lateral walking direction (X-axis).

To be able to determine the start of the speed adjustments, we first had to establish in which scenarios absolute speeds decreased to a sufficient extent as compared to the negligible speed fluctuations in the No-interferer scenario. On that account, we identified the lowest local minima (minimum speed) and the absolute maxima (maximum speed) within each trial of each scenario. It was further required that the minimum speed had to be surrounded by two speed peaks to exclude a minimum speed that occurred in the very beginning or the very end of a trajectory. The difference between the minimum and maximum speed was calculated (*speed change*) and gives information about whether meaningful speed adjustments occurred.

Similar to the start of turning, the start of speed adjustment (hereafter called *start of braking*) was calculated as the spatial distance between the first point along the walking direction where the speed started to decrease below the baseline speed and PoMD. The baseline speed was defined as the mean absolute speed in the No-interferer scenario subtracted by two standard deviations, calculated separately for each participant and each speed condition. An example of the start of braking is printed as×in Fig. 4.

#### 4.5 Statistical analysis

Statistical analysis was calculated using Matlab. The normality assumption was checked using the Lilliefors test. For all conditions, the normality assumption could not be rejected. A one-way repeated measurement ANOVA was calculated with the two between-group factors *scenario* and *speed* for the following parameters: mean absolute speed, maximal lateral deviation, start of turning, speed change, and start of braking. Post-hoc analysis was calculated using Tukey HSD for the significant main effects and additionally, partial one-way ANOVA’s were calculated for post-hoc analysis of significant interactions. The critical value of statistical significance was set at *p* = 0.05.

Data from 11 out of 900 trials (1.2%) had to be discarded from the statistical analysis due to insufficient valid data points as a result of obstructed markers during recording. These invalid trials encompassed 1 trial in the slow speed condition, 8 in the normal speed condition and 2 in the fast speed condition.

## Results

In the following, the main results in terms of path and speed adjustments are reported. The mean trajectories for all scenarios and conditions as well as the start of turning and the start of braking are plotted in Fig. 5. To begin with, test results on whether the speed conditions showed significant changes in their mean velocities will be presented. This analysis was done to verify that the participants moved according to the instructions.

### 1 Speed Condition Validation

We analyzed the differences in mean absolute speed amongst speed conditions to confirm that participants walked at different speeds as instructed, and to examine potential speed differences amongst scenarios. Overall, the results show that the participants moved with different velocities according to the instructions, as revealed by the significant main effect of speed, *F*(2,90) = 40.90, *p*<0.001. Post-hoc comparisons showed that the mean walking speed differed amongst all three speed conditions (means for slow: 1.15 m/s, normal: 1.42, and fast: 1.78 m/s), all *p’*s <0.001.

### 2 Path Parameters

#### 2.1 Lateral deviation from the straight path

**Figure 6:**
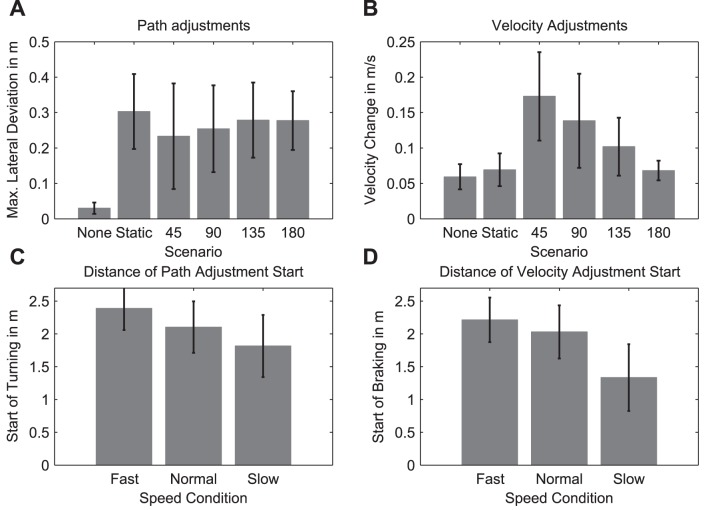
Plot of the relevant parameters. **A:** The maximal lateral deviation from the movement direction for each crossing scenario, averaged across all walking speeds. All scenarios show a lateral deviation different from the No-interferer scenario, but not different from each other. **B:** The speed change for each crossing scenario, averaged across all walking speeds. Only the 45° and 90° crossing scenarios are significantly different from the No-interferer scenario. **C:** The start of turning (i.e. start of path adjustments) for each walking speed condition, averaged across all scenarios. For a higher speed, the path adjustment occurs at larger distances to PoMD. **D:** The start of braking (i.e. start of speed adjustments) for each walking speed condition, averaged across all scenarios. When walking at a speed slower than normal, braking is initiated closer to PoMD than when walking at normal or faster than normal speed.

Participants made a spatial deviation in their path whenever there was an interferer, as revealed by the significant main effect of scenario, *F*(5,45) = 21.87, *p*<0.001, and subsequent post-hoc analysis (see Fig. 5 & 6). No other scenario was found to differ from the others. As a consequence, all scenarios where the interferer was present were passed to further statistical analyses related to path adjustments. Neither the main effect of speed nor the interaction of scenario and speed reached the level of significance.

#### 2.2 Start of turning

The start of turning was significantly influenced by the crossing angle between the participant and the interferer (main effect of scenario: *F*(4,36) = 25.15 *p*<0.0001), as well as by the walking speed (main effect of speed: *F*(2,18) = 48.35 *p*<0.0001). For the main effect of scenario, post-hoc comparisons revealed that turning started closer to PoMD in the 45° and 90° scenarios than in the 135° scenario, and in the static and 180° scenarios (see Table 1), all *p*’s<0.05, while the former and the latter groups did not differ amongst themselves. Post-hoc tests related to the main effect of walking speed identified that the start of turning was significantly different amongst all three speeds, such that it occurred furthest to PoMD in the faster speed condition and closest to PoMD in the slower speed condition (see Table 1, and Fig. 5 & 6).

**Table 1:** The table shows at which distance to the point of minimal distance (PoMD; in meter) the adjustments of path and speed were initiated.

Parameter	speed condition	static	45°	90°	135°	180°	scenariodependent	speeddependent
**Start of turning**	***fast***	2.5	2.2	2.2	2.4	2.7	Yes	Yes
	***normal***	2.4	1.7	1.9	2.1	2.4		
	***slow***	2.4	1.5	1.5	1.7	2.1		
**Start of braking** *	***fast***	/	2.3	2.1	/	/	No	Yes
	***normal***	/	1.9	2.1	/	/		
	***slow***	/	1.3	1.4	/	/		

*For the start of braking, only values for the 45° and 90° scenarios are reported, as relevant adjustments of speed consistently occurred only for these two scenarios.

The interaction between scenario and speed was also significant (*F*(8,72) = 11.42, *p*<0.0001). Post-hoc analysis revealed a significant main effect of scenario in the faster (*F*(4, 36) = 19.81, *p*<0.01), and slower speed conditions (*F*(4, 36) = 7.29, *p*<0.05). In the faster speed condition, the point of first turning occurred closer to PoMD in the 90° scenario than in the 180° scenario (see Table 1). In the slower speed condition, the point of first turning occurred closer to PoMD in the 45° scenario than in the static and 180° scenarios (see Table 1), all *p’*s <0.05.

Through additional calculations we found that some of the obtained point of first turning coincided with the first recorded data point. This suggests that the obtained values do not represent the actual point of first turning, which may have occurred even earlier, before participants entered the tracked area. However, regarding the results, this methodological issue may have rather *reduced* the observed effect, especially between the faster and the other speed conditions. This implies that the real difference between the speeds may have been even greater, had we have been able to track the entire walking distance.

### 3 Speed Parameters

#### 3.1 Speed changes

Significant speed adjustments were found in three scenarios, as revealed by the significant main effect of scenario (*F*(5, 45) = 28.52, *p*<0.001) and subsequent post-hoc analysis. Thereby, post-hoc comparisons identified that the speed changes in the 45°, 90°, and 135° scenarios were significantly higher than in the No-interferer, static, and 180° scenarios, all *p’*s<0.001, while the latter three scenarios did not differ from one another. The same post-hoc test also revealed that the speed change in the 45° scenario was significantly greater than in the 90° scenario, which in turn was greater than in 135° scenario, all *p’*s <0.05. In addition, speed changes were significantly influenced by the walking speed (*F*(2, 18) = 22.10, *p*<0.001), such that greater speed changes occurred in the slower speed condition than in the normal and faster speed conditions.

The interaction between scenario and speed also reached the level of significance, *F*(10, 90) = 3.21, *p*<0.01. Partial one-way ANOVA’s for each speed revealed the following:

1. A significant main effect of scenario for the normal speed condition, *F*(5, 45) = 35.34, *p*<0.001. Post-hoc tests showed that the speed changes in the 45° and 90° scenarios were significantly greater than in the No-interferer, static, 135°, and 180° scenarios, all *p’*s<0.001, while the former and latter group did not significantly differ amongst themselves.
2. For the slower speed condition, the main effect of scenario was also significant, *F*(5, 45) = 21.66, *p*<0.01. Post-hoc tests showed that the speed changes in the 45°, 90°, and 135° scenarios were significantly greater than in the No-interferer, static, and 180° scenarios, whereas the latter three did not differ between one another. In addition, the speed change in the 45° scenario was significantly greater than in the 135° scenario.
3. For the faster speed condition, the main effect of scenario approached the level of significance, *F*(5, 45) = 4.67, *p* = 0.058. Post-hoc tests showed that only the speed change in the 45° scenario was significantly greater than in all the other scenarios, which did not differ amongst themselves.

As meaningful speed changes most consistently occurred in the 45° and 90° scenarios, only these two conditions will be considered in further analyses of speed adjustments.

#### 3.2 Start of braking

Having established in which scenarios speed changes occurred; it was then of interest where participants started reducing their speed as part of the collision avoidance strategy. The results show that when participants walked in the slower speed condition, they also started braking later in the trajectory (i.e. closer to PoMD) than in the normal and faster speed conditions (see Table 1), as revealed by a significant main effect of speed (*F*(2,2) = 21.87 *p*<0.001) and subsequent post-hoc analysis. Neither the main effect of scenario nor the interaction between the two factors reached the level of significance.

### 4 Relation between Path and Speed Adjustments

In order to determine whether and how the path and speed adjustments were related to each other, we calculated the mean difference between the start of the path adjustment and the start of the speed adjustments per participant for the 45° and 90° scenarios, across the different speed conditions. A t-test did not reveal differences in the spatial position of the initiation of path and speed adjustments.

## Discussion

In this study, we investigated whether and where path and speed adjustments (i.e., turning and braking, respectively) were initiated to avoid collision when participants crossed another person at different angles and walking speeds. Furthermore, we investigated whether the potential path and speed adjustments were jointly or independently initiated. The following discussion is organized according to these research questions. At the end of this part, we aim to compare our results with principle model predictions.

### 1 Do Pedestrians Adjust both Walking Path and Speed to Avoid Collisions?

To answer this question, we analyzed maximal lateral deviation from the straight path and the velocity change between different crossing angles and walking speeds. On that account, two crossing scenarios (static & 180°) that required an adjustment of the walking path were implemented, whereas in the other scenarios adjustment of either one of the two parameters could have led to successful collision avoidance. As a general result, we found that path and speed are differently adjusted with respect to different crossing scenarios and walking speeds.

We first aimed to verify whether path adjustments represent a preferred collision avoidance strategy as opposed to speed adjustments. As the interferer’s behavior was designed to create a hypothetical collision at the same spatial position in all the experimental conditions, in principle each trajectory that allowed passing a static interferer without collision would have been a successful strategy for all the other scenarios, too. That is, participants could have planned their strategies globally, as we actually assume for the static scenario in line with previous findings [2], for all the scenarios. However, our results show that the use of path and speed adjustments varied amongst scenarios, which indicates that the walking trajectories were locally adjusted according to the interferer’s crossing angle. This finding is in line with recent work by our group suggesting a local planning in the presence of a moving interferer [1]. Specifically, path adjustments were observed in all the scenarios where an interferer was present, whereas speed adjustments were evident only in scenarios with a crossing angle of 45° and 90° (see Fig. 6A & B). Furthermore, path adjustments seemed to be independent of the walking speed, whereas speed adjustments were influenced by different walking speeds (see Table 1). Thus, it appears that path adjustments were generally applied as a collision avoidance strategy, whereas braking was applied only under certain circumstances which will be discussed below.

### 2 How are Walking Path and Speed Influenced by the Crossing Angle and Walking Speed?

We found that only the walking path, but not the walking speed, was adjusted in the two scenarios that required path adjustments for successful collision avoidance (static and 180° scenario). This is in partial accordance with previous findings, showing only path adjustments for a static obstacle, while both path and speed adjustments were found when crossing at 180° [5]. Furthermore, our results are in line with the predictions of the smoothness-optimization model of locomotion [1] [13], and are even more consistent with an advanced model of this theory, which adds an extra term that penalizes large variations of the speed [14]. As speed variations are penalized, turning should clearly be a favored strategy. This is indeed what was observed in our study. Participants were given sufficient distance and time prior to the possible collision with the interferer, so they could freely choose their avoidance strategy as well as when and where they initiated it. As a consequence, participants chose a collision avoidance strategy of smooth spatial adjustments (see Fig. 5), which was initiated early enough to render speed adjustments unnecessary.

In the other crossing scenarios, on the contrary, both path and speed adjustments were observed (see Fig. 6A & B), although there would have been alternative successful strategies, e.g. adjusting only the speed while maintaining a straight path [1]. This result again supports the assumption of path adjustments as a default collision avoidance strategy in the presence of sufficient space. Our results further suggest that crossing another moving person at acute angles, such as 45° and 90°, poses additional demands on the navigation behavior, and that successful collision avoidance in these situations entails more complex strategies, namely the adjustment of movement path *and* speed. The question thus arises: Why does crossing at these acute angles impose higher demands, such that both path and speed adjustments were required to avoid collision? Two explanations may account for that:

#### 2.1 Constraints imposed by the interferer in the 45° and 90° scenarios

**Figure 7:**
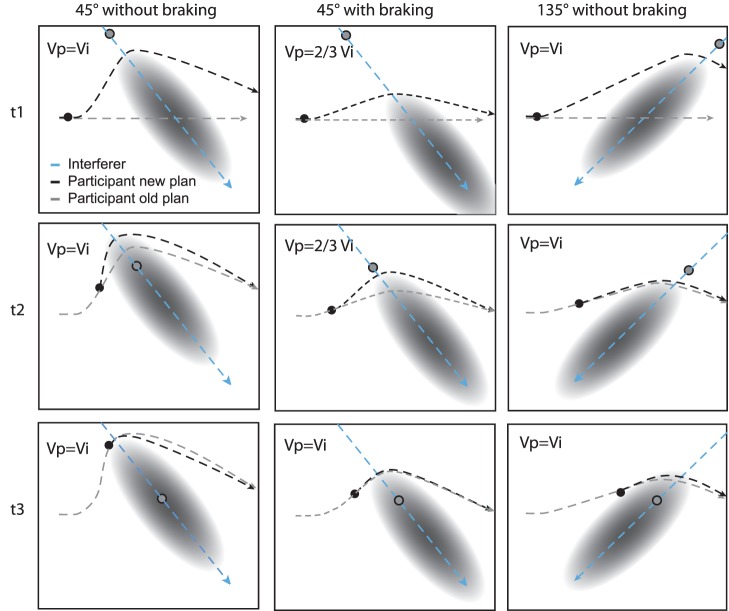
Schematic plot of the planned trajectories to avoid a hypothetical collision. All scenarios start at the same position at time t1, the pedestrian (gray dot) and interferer (black dot) have the same speed (Vi = Vp) and the same distance to the point of crossing, similar to our set-up. The originally planned trajectories for both the pedestrian and interferer are represented as gray dashed arrows, whereas each dash represents the distance travelled in one time step. The hypothetical collision is predicted with some uncertainty, which is stretched along the interferer’s movement direction (gray ellipse). A new path (black dashed line) is planned every three time steps. The middle and last row show the situation after 3 (t2) or 6 time steps (t3), respectively. Each row illustrates another collision avoidance strategy. **Left:** The planned trajectory (black dashed line) if only turning is considered in the 45° scenario. High angular velocities must be applied to turn around the obstacle. **Middle:** The planned trajectory for the 45° scenario if the pedestrian reduces his speed to 2/3 of the speed of the interferer at time t1. As soon as the pedestrian can freely pass the obstacle without a collision, the speed is set to the original speed again (t3). The combination of small speed and path adjustments allows a smooth trajectory. **Right:** The planned trajectory for the 135° scenario. The pedestrian and interferer have the same speed. A smooth path can be achieved without a speed adjustment.

Models based on repulsive potentials [11] [12], uncertain collision estimates [1], and recent findings of personal spaces [18], suggest the existence of additional spatial constraints around the interferer. These “interferer constraints” are assumed to be spread mainly towards the walking direction (i.e. not spherical, see Fig. 7). Furthermore, there is ample empirical evidence that pedestrians plan their trajectories in a predictive manner, i.e., they estimate potential collisions with moving obstacles and adjust their trajectories accordingly [1] [16]. This concept is implemented in recent models for crowd simulations [7] [19] [20], computer graphics and games [12] [21], and mobile robot navigation, e.g. the speed obstacle approach [22]. The above mentioned constraints have less influence in scenarios where the interferer is crossing at obtuse angles. However, at crossing angles of 45° or 90°, these constraints might become relevant.

To illustrate the spatial constraints at acute angles (see Fig. 7), we plotted the temporal development of the walking trajectory for the 45° (1^st^ and 2^nd^ column) and the 135° (3^rd^ column) scenario at three different time points. Note that it represents a schematic plot the walking trajectories based on the assumptions of predictive trajectory planning and spatial constraints around the participant and interferer, and not a numerical simulation of the data. For the 45° scenario, we depict two different strategies, one where only turning is applied (1^st^ column) and a second strategy where turning and braking to 2/3 of the original speed (2^nd^ column) are applied. For the 135° scenario, only the movement path is adjusted, as was observed in our study. In the 45° scenario, crossing the other pedestrian without adjusting walking speed would require substantial changes in the movement path (see Fig. 7, 1^st^ column, 1^st^ row). The same strategy would result in much smoother obstacle avoidance in the 135° scenario (Fig. 7, 3^rd^ column, 1^st^ row). However, an even smoother circumvention of the obstacle can be achieved in the 45° scenario, if movement speed is adjusted in addition to movement path (see Fig. 7, 2^nd^ column, 1^st^ row). Since it is assumed that the walking trajectory is continuously planned and updated [15], after a certain time (2^nd^ and 3^rd^ row) the predicted position of the obstacle has moved further along the path, and a potential collision becomes less likely. This then allows for a more direct path towards the goal (2^nd^ row). From a certain time point on (see 3^rd^ row), it becomes clear that there will be no collision when heading directly to the goal. In addition, when applying turning and braking in the 45° scenario, the walked path has a lateral deviation that is comparable with the 135° scenario, which is in line with our findings (see Fig. 5, 3^rd^ & 5^th^ row; Fig. 7, 3^rd^ row). Without braking the lateral deviation would be much greater (see Fig. 7, 3^rd^ row). The results thus suggest that pedestrians can optimize smoothness criteria at acute crossing angles by implementing braking, thereby avoiding big changes in the walking path. Besides, sharp turnings, as they would have been necessary without a decrease in movement speed, would have been carried out under time pressure and would have required a less favorable steering mechanism (i.e., led by hip motion instead of foot placement) [23]. This would potentially make the locomotion less stable. As such, participants in 45° and 90° scenarios made the spatial collision avoidance strategy more economical by additionally adjusting their movement speed.

#### 2.2 Lower relative speed in the 45° and 90° scenarios

The second plausible reason for the ‘turning plus braking’ strategy is associated with the possible control mechanism underlying this strategy (a more detailed discussion on that see 3.1). The initiation of both path and speed adjustment seems to be based on the perceived time to a hypothetical collision (TTC, [23], see also 3.1). Approximating TTC requires the extraction of relevant spatio-temporal information, which could be best derived from the relative walking speed of the interferer – that is, the speed difference between the two pedestrians, taking into account their directional difference. As such, the speed of the interferer relative to the participant in the 45° or 90° scenarios was lower than that in the 180°. With a lower relative speed, the estimation of TTC became more difficult and less accurate, as the potential error in approximating the interferer’s speed became larger in proportion to the relative speed itself. Confronted with greater difficulty in the estimation, participants in these scenarios therefore had to employ a reduction in speed to gain more time for the online calculation of TTC [9]. This interpretation is supported by the observation that turning was initiated closer to PoMD in the 45° and 90° scenarios than in the static and 180° scenarios. This indicates that, due to the more difficult estimation of TTC, participants were less certain with the initiation of their strategies in 45° and 90°, and they thus delayed their adjustment. Delaying the initiation of spatial adjustment also made a decrease in speed necessary (as speculated in the first explanation) in order to avoid collision.

Still other factors may have accounted for the difference between the ‘turning only’ strategy in the static and 180° scenarios, and the ‘turning plus braking’ strategy in the 45° and 90° scenarios. As an example, visual information is often found to guide locomotion [23], and one might speculate that the available visual information differed across scenarios for the spatial movement planning. Presumably, in the static and 180° scenarios, and perhaps also in the 135° scenario, participants received more complete visual information of the interferer’s movement in their central vision than they did in 45° and 90° scenarios, and this might have contributed to easier planning of the adjustments in the former scenarios. This interpretation is, however, less supported by previous findings. For example, Marigold and colleagues [25] found that central vision is not a prerequisite in avoiding unexpected obstacles, and peripheral vision works just as well for that purpose. Besides, as head rotation was not prohibited in the present task, participants were free to turn their head to gather necessary information for navigation. As head rotation has been found not to influence the turning behavior in the trajectory [26], we can safely assume our participants had turned their head to gather visual information in the 45° and 90° scenarios, and the head turning itself did not affect the outcome of their adjustment. Therefore, it seems more plausible that the complex strategy observed in these scenarios is a result of the two aforementioned reasons.

### 3 What Control Mechanism Underlies the Path and Speed Adjustments?

There are different attempts to describe the navigation behavior of pedestrians in the presence of static or moving obstacles. In the following sections, we will compare principle predictions of different models with our experimental data. We will compare between the predicted and the observed patterns with regard to the spatial positions where path and speed adjustments are initiated, as well as the obstacle position used to estimate this initiation.

#### 3.1 Initiation of path and speed adjustments

Our results are in contrast to a number of models of human obstacle avoidance behavior with respect to two aspects: whether path and speed adjustments are jointly initiated, and where they are initiated. Regarding the first aspect, our results speak against a two-stage planning of walking path and speed, where either turning [7] [9] or braking [10] is initiated first. The observed differences may be attributed to the different spatial or visual constraints imposed in these studies.

Regarding the second aspect, different predictions have been made depending on the model. One of the first models that tried to explain the dynamics of pedestrians was the “social force model” [11]. This model predicts the initiation of path and speed adjustments to be closer to the obstacle for higher pedestrian velocities (see Fig. 1A). Another model from Fajen and Warren [3] modeled the behavior of a pedestrian as a dynamical system in which angular acceleration is a function of three variables: goal, obstacle angle, and distance. This model does not take into account the pedestrian’s speed; therefore, where path adjustment is initiated should not depend on the speed conditions (see Fig. 1A). An advancement of this model [4] tries to account for different walking speeds, though it is not capable to explaining the coordination between path and speed adjustments [27]. A recent model based on heuristic rules [7] uses a “horizon distance”, which is a fixed spatial value. The path of the pedestrian is then adjusted in a way that allows for the most direct path to the destination, taking into account the presence of obstacles. As second rule in this model, speed adjustments are employed to keep a safety distance, which is TTC-controlled. Therefore, adjustments of path should be initiated independent of the pedestrian’s speed (see Fig. 1B). In addition, according to the parameters suggested by the model, path adjustments should occur far before speed adjustments. Finally, another principle to initiate collision avoidance adjustments is the estimate of the TTC [15] [24]. Faster moving pedestrians should adjust path and speed at a larger distance to a hypothetical collision point than slower moving ones, as the initiation of strategies is based on the time estimated to reach the obstacle. Although there is still ambiguity about which behavioral variables are used to estimate TTC [15], a number of recent models account for this principle [4] [27].

Our data clearly provides evidence for a control mechanism where both path and speed adjustments are based on the same constantly updated estimation of TTC (see also Fig. 7 for a schematic illustration). We show that, when both spatial and speed adjustments are applied, such as in our 45° and 90° scenarios, there is a relation between the initiation of turning and the initiation of braking. This relation is revealed by the following results: First, both the ‘start of turning’ and the ‘start of braking’ depend on participants’ walking speed, i.e. the faster walking speed, the further away from PoMD participants initiate turning and braking (see Fig. 6C & D). Secondly, the initiation of both did not differ from each other in space. Together, it suggests that the initiation of both parameters is based on the same behavioral variable; it also suggests that if the pedestrian decides to initiate an obstacle avoidance maneuver, path and speed are simultaneously controlled. This result is not unreasonable, as the estimated time to collision is a highly relevant variable to ensure there is enough time for passing an interferer in a smooth way. Finally, the start of turning was influenced by the crossing scenario in such a way that turning was initiated closer to PoMD at acute crossing angles than in the other scenarios. As briefly mentioned in section 2, this finding may be explained by the more difficult estimation of TTC in scenarios with acute crossing angles.

In sum, we propose that the TTC principle is suitable for modeling navigation behavior of pedestrians, whereby adjustments of path and speed are based on the same behavioral variables to estimate TTC. While it is possible to modify heuristic models to be compatible with the TTC, the same is problematic with models based on repulsive potentials. To our knowledge, an implementation of a TTC behavior with repulsive potentials is not possible. We therefore conclude that models based on repulsive potentials principle (e.g. [11] [12]) are not suitable for explaining general human locomotion and collision avoidance behavior.

#### 3.2 Are the dynamics of the obstacle considered to avoid a collision?

The above-mentioned models have specific assumptions about the obstacle position that is used to estimate the initiation of the obstacle avoidance behavior. Models developed for static obstacles naturally use the position of the obstacle to avoid a collision [3]. However, if the obstacle is moving, two different approaches can be found in the literature that deal with the position of the obstacle:

1. Similar to a static obstacle, a penalty region around the actual position of the moving obstacle is assumed [3] [22]. To account for the fact that pedestrians require space for the next step, this penalty region may be shifted along the moving direction [11]. In the context of our study, this approach would predict that people start to adjust their path earlier in the static than in the 180° scenario, since the interferer is much closer in the static scenario.
2. The second approach assumes the estimation of a hypothetical collision point in the future [7] [12]. This requires an internal model that contains assumptions of the dynamics of the environment, which is in our case how the path of the interferer and of the pedestrians themselves will develop in time. The simplest model is the linear extrapolation of the trajectory with constant direction and speed [7] [12]. Based on that, there should be no difference in the initiation of the path adjustment for the static and 180° scenario, since the point of hypothetical collision is the same in both situation.

A comparison of the start of path adjustments in the 180° and in the static scenario revealed that there is no difference in the start position. Thus, our results provide evidence for the second strategy, that pedestrians consider the dynamics of the interferer to predict a hypothetical collision, and they avoid this position. However, we suggest that pedestrians also consider the uncertainty about a hypothetical collision. We assume a higher uncertainty along the speed parameter of the obstacle, as this would explain the braking behavior for acute crossing angles. A recent model by Fajen [27] accounts for the dynamics of the interferer and TTC. Though, at its current stage it is only able to describe obstacle avoidance behavior when pedestrians and obstacles move at constant velocity (i.e. speed and movement direction). Given that it can be expanded to changing movement velocities, this model may be capable of describing our results.

### 4 Conclusion

We investigated the collision avoidance behavior of pedestrians walking at different speeds in the presence of a non-reactive interferer crossing at different angles. We focused on the adjustment of movement path and speed as parameters of the applied strategies, which were found to depend on both crossing angle and walking speed. We conclude that the trajectories are locally planned and adjusted according to the hypothetical collision with the interferer. Furthermore, we found path adjustments for all the crossing angles, while speed adjustments were present only in scenarios where the interferer was crossing at acute angles. In the acute angle scenarios, both path and speed adjustments were initiated via the same TTC estimate. We suggest that TTC estimation allows the pedestrian to pass the interferer with a smooth trajectory. The present study can be used to evaluate and adjust existing pedestrian dynamics models or serve as empirical basis to develop new models.

## References

[1] Basili P, Sağlam M, Kruse T, Huber M, Kirsch A, et al (2013) Strategies of locomotor collision avoidance. Gait Posture 37(3): 385–390. 22975461 10.1016/j.gaitpost.2012.08.003

[2] Hicheur H, Pham QC, Arechavaleta G, Laumond JP, Berthoz A (2007) The formation of trajectories during goal-oriented locomotion in humans. I. A stereotyped behavior. Eur J Neurosci 26(8): 2376–239. 17953625 10.1111/j.1460-9568.2007.05836.x

[3] Fajen BR, Warren WH (2003) Behavioral dynamics of steering, obstacle avoidance, and route selection. J Exp Psychol Hum Percept Perform 29(2): 343–362. 12760620 10.1037/0096-1523.29.2.343

[4] Fajen BR, Warren WH (2007) Behavioral dynamics of intercepting a moving target. Exp Brain Res 180(2): 303–319. 17273872 10.1007/s00221-007-0859-6

[5] Moussaid M, Helbing D, Garnier S, Johansson A, Combe M, et al (2009) Experimental study of the behavioural mechanisms underlying self-organization in human crowds. Proc Biol Sci. 276(1668): 2755–2762. 10.1098/rspb.2009.0405 PMC2839952 19439442

[6] Jansen SE, Toet A, Werkhoven PJ (2011) Human locomotion through a multiple obstacle environment: strategy changes as a result of visual field limitation. Exp Brain Res 212(3): 449–456. 21687987 10.1007/s00221-011-2757-1 PMC3127014

[7] Moussaid M, Helbing D, Theraulaz G (2011) How simple rules determine pedestrian behavior and crowd disasters. Proc Natl Acad Sci U S A 108(17): 6884–6888. 21502518 10.1073/pnas.1016507108 PMC3084058

[8] Cinelli CE, Patla AE (2008) Task-specific modulations of locomotor action parameters based on on-line visual information during collision avoidance with moving objects. Hum Mov Sci. 27(3): 513–531. 10.1016/j.humov.2007.10.001 18234382

[9] Cinelli ME, Patla AE (2008) Locomotor avoidance behaviours during a visually guided task involving an approaching object. Gait Posture 28(4): 596–601. 18514525 10.1016/j.gaitpost.2008.04.006

[10] Olivier AH, Marin A, Crétual A, Berthoz A, Pettré J (2013) Collision avoidance between two walkers: Role-dependent strategies. Gait Posture 38(4): 751–756. 23665066 10.1016/j.gaitpost.2013.03.017

[11] Helbing D, Molnar P (1995) Social force model for pedestrian dynamics. Phys Rev E Stat Phys Plasmas Fluids Relat Interdiscip Topics 51(5): 4282–4286. 9963139 10.1103/physreve.51.4282

[12] Karamouzas I, Heil P, van Beek P, Overmars MH (2009) A predictive collision avoidance model for pedestrian simulation. In: Egges A, Geraerts R, Overmars M, editors: Motion in Games 2009, Springer-Verlag Berlin. 41–52.

[13] Pham QC, Hicheur H, Arechavaleta G, Laumond JP, Berthoz A (2007) The formation of trajectories during goal-oriented locomotion in humans. II. A maximum smoothness model. Eur J Neurosci 26(8): 2391–2403. 17953626 10.1111/j.1460-9568.2007.05835.x

[14] Pham QC, Hicheur H (2009) On the Open-Loop and Feedback Processes That Underlie the Formation of Trajectories During Visual and Nonvisual Locomotion in Humans. J Neurophysiol 102(5): 2800–2815. 19741106 10.1152/jn.00284.2009

[15] Tresilian JR (1999) Visually timed action: time-out for “tau?”. Trends Cogn Sci 3(8): 301–310. 10431184 10.1016/s1364-6613(99)01352-2

[16] Olivier AH, Marin A, Crétual A, Pettré J (2012) Minimal predicted distance: A common metric for collision avoidance during pairwise interactions between walkers. Gait Posture 36(3): 399–404. 22560717 10.1016/j.gaitpost.2012.03.021

[17] Hicheur H, Vieilledent S, Richardson MJE, Flash T, Berthoz A (2005) Velocity and curvature in human locomotion along complex curved paths: a comparison with hand movements. Exp Brain Res 162(2): 145–154. 15586276 10.1007/s00221-004-2122-8

[18] Gérin-Lajoie M, Richards CL, Fung J, McFadyen BJ (2008) Characteristics of personal space during obstacle circumvention in physical and virtual environments. Gait Posture 27(2): 239–247. 17512201 10.1016/j.gaitpost.2007.03.015

[19] Paris S, Pettré J, Donikian S (2007) Pedestrian reactive navigation for crowd simulation: a predictive approach. Comput Graph Forum 26(3): 665–674.

[20] Hoogendoorn SP (2004) Pedestrian flow modeling by adaptive control. Transportation Research Record: Journal of the Transportation Research Board 1878(1): 95–103.

[21] Reynolds CW (1999) Steering behaviors for autonomous characters. Proceedings of the Game Developers Conference: 763–782.

[22] Fox D, Burgard W, Thrun S (1997) The dynamic window approach to collision avoidance. IEEE Robotics and Automation Magazine 4(1): 23–33.

[23] Patla AE, Adkin A, Ballard T (1999) Online steering: coordination and control of body center of mass, head and body reorientation. Exp Brain Res 129(4): 629–634. 10638436 10.1007/s002210050932

[24] Cinelli ME, Patla AE (2007) Travel path conditions dictate the manner in which individuals avoid collisions. Gait Posture 26(2): 186–193. 17049236 10.1016/j.gaitpost.2006.08.012

[25] Marigold DS, Weerdesteyn V, Patla AE, Duysens J (2007) Keep looking ahead? Re-direction of visual fixation does not always occur during an unpredictable obstacle avoidance task. Exp Brain Res 176(1): 32–42. 16819646 10.1007/s00221-006-0598-0

[26] Cinelli M, Warren WH (2012) Do walkers follow their heads? Investigating the role of head rotation in locomotor control. Exp Brain Res 219(2): 175–190. 22466410 10.1007/s00221-012-3077-9 PMC3592975

[27] Fajen BR (2013) Guiding locomotion in complex, dynamic environments. Front Behav Neurosci 7: 85. 23885238 10.3389/fnbeh.2013.00085 PMC3716022

